# Risk Factor Analysis for Breast Cancer in Premenopausal and Postmenopausal Women of Punjab, India

**DOI:** 10.31557/APJCP.2019.20.11.3299

**Published:** 2019

**Authors:** Akeen Kour, Sarika Sharma, Vasudha Sambyal, Kamlesh Guleria, Neeti Rajan Singh, Manjit Singh Uppal, Mridu Manjari, Meena Sudan, Sahiba Kukreja

**Affiliations:** 1 *Human Cytogenetics Laboratory, Department of Human Genetics, Guru Nanak Dev University, *; 2 *Department of Surgery, *; 3 *Department of Pathology, *; 4 *Department of Radiotherapy,*; 5 *Department of Biochemistry, Sri Guru Ram Das Institute of Medical Sciences and Research, Vallah, Amritsar, Punjab, India. *

## Abstract

**Objective:**

Amritsar, the second largest town of agrarian state of Punjab, India reports high number of breast cancer cases every year. The present study investigated the etiology of breast cancer using various obesity indices and other epidemiological factors among breast cancer patients residing in and around Amritsar city.

**Methods:**

In this case control study, risk factors for breast cancer were analyzed in 542 female subjects: 271 females with breast cancer patients and 271 unrelated healthy females matched for age as control females.

**Results:**

Bivariate analysis for risk factors in cases and controls showed a lower risk (OR=0.65, 95% CI 0.43-0.99, p=0.04) in obese cases with BMI≥25kg/m^2^ as compared to subjects with normal BMI. Risk factor analysis showed that parameter which provided risk for cancer in postmenopausal women was obesity and in premenopausal women was parity. Postmenopausal women with BMI (overweight: OR=0.39, 95% CI 0.17-0.92, p=0.03; obese: OR= 0.26, 95% CI 0.13-0.52, p=0.00), WC (OR=0.17, 95% CI 0.05-0.52, p=0.00) and WHtR (p=0.02) had highr risk. Premenopausal women with 3 or less than 3 children had a higher risk (OR=5.54, 95 % CI 2.75-11.19, p=0.00) than postmenopausal women when compared to women with more than 3 children. Binary logistic regression analysis revealed that low parity (≤3) substantially increased the risk for breast cancer (OR=4.80, 95% CI 2.34-9.85, p=0.00) in premenopausal women.

**Conclusion:**

Obesity, parity associated breast cancer risk and reduced breastfeeding cumulatively predispose the premenopausal women of this region to higher risk of breast cancer.

## Section Title

Breast cancer, risk factors, parity, obesity, premenopausal, postmenopausal

## Introduction

Breast cancer, a genetically and histopathologically heterogeneous disease, is leading cancer in Asian females (Grayson, 2012). In India, among males and females, number of breast cancer patients is expected to cross the figure of 100,000 by the year 2020 (Takiar et al., 2010). Various risk factors including early menarche, late menopause, late first full-term pregnancy, nulliparity, no breast feeding, family history of breast cancer, obesity in postmenopausal women, hormone replacement therapy, exposure to low dose radiation, have been reported to be associated with breast cancer (Parmar and Cunha, 2004; Althuis et al., 2005; Ma et al., 2006; Yeole 2008, Collaborative Group on Hormonal Factors in Breast Cancer, 2012). 

Obesity has been considered as one of the most important known preventable causes of cancer (Haslam and James, 2005) which has a complex relation to risk of breast cancer. Anthropometric indicators such as body mass index (Van Den Brandt et al., 2000; Palmer et al., 2007) and waist circumference (Palmer et al., 2007; Huang et al., 1997; Borugian et al., 2003) have been highly associated with breast cancer risk. Malignant proliferation of breast tissue in women has also been associated with changes in plasma lipid and lipoprotein level (Lane et al., 1995). Low levels of high density lipoprotein (Furberg et al., 2004), high blood glucose (Sieri et al., 2013) and high triglycerides (Potischman et al., 1991; Kapil et al., 2013) have been associated with breast cancer. 

In India, breast cancer has been reported as the second most common form of malignancy among women in states of Punjab, Maharashtra and Gujarat (Ali et al., 2011). In Punjab, a state in North West India, increasing cancer incidence is being reported from its different regions i.e. Majha, Malwa and Doaba (Thakur et al., 2008), though etiology of cancer in state is not completely understood. Punjab also has higher incidence of obesity whose relation with cancer etiology has not been thoroughly investigated. According to National family health survey (NFHS-4) Punjab, India, 2015-2016; 27.8% men and 31.3% women in Punjab are overweight or obese. A recent report from India’s Department of Health and Family Welfare (DHFW, 2013) stated the cancer prevalence (per million) in the Majha region of Punjab as 647 as compared to India’s national average cancer prevalence of 800/million. Amritsar in the Majha region of Punjab is the second largest city of Punjab. Its population is 11.2 lakhs (2011 Census Data). The frequency of sporadic breast cancer is increasing in areas adjoining Amritsar city of Punjab (Batra et al., 2010). The present study investigated the etiology of breast cancer using various obesity indices and other epidemiological factors among breast cancer patients residing in and around Amritsar city of Punjab (India).

## Materials and Methods

This study was conducted after approval by the institutional ethical committee of Guru Nanak Dev University, Amritsar,Punjab, India. In this case control study, risk factors for breast cancer were analyzed in 542 female subjects: 271 breast cancer patients and 271 unrelated healthy females matched for age as controls. Patients with confirmed malignant breast cancer without any history of any other cancer were included in the study whereas patients who had received blood transfusion, prior therapy (chemotherapy, hormone therapy, radiotherapy or surgery) were excluded from the study. The patients were clinically investigated at Sri Guru Ram Das Institute of Medical Sciences and Research, Vallah, Amritsar, Punjab, India. Two hundred and seventy one age and gender matched unrelated healthy control individuals having no past history of any cancer were included in the study. The control subjects were residents of Punjab randomly selected from the same geographical area as cancer patients. The controls recruited in the present study were residents of the villages, from various districts of Punjab adjoining Amritsar city. Before data collection, the Sarpanch (Headman) of the village was contacted who provided the list of adult residents, both men and women. Among these, subjects who were interested to participate in the current study were randomly selected and an attempt was made to enroll a single woman from each nuclear family. Women belonging to age group 25-70 years were recruited as controls.

Subjects suffering from any chronic disease for at least one year prior to date of sampling were excluded from the study. Relevant information including age, gender, occupation, personal history, habitat, habits and disease history were recorded in pre-designed questionnaire. Anthropometric measurements including height, weight, waist circumference and hip circumference were taken using standard anthropometric techniques (Singh and Bhasin, 1968; Weiner and Lourie, 1981). Individuals were classified according to Body Mass Index (BMI) using WHO criteria for Asian population (WHO, 2004). Cut off values recommended for Asian population; for waist circumference as 80 cm for women and waist-hip ratio as 0.81 for women (Snehalatha et al., 2003) were used for the assessment of abdominal adiposity. 

Data thus obtained was analyzed by chi-square test and binary logistic regression using SPSS (Version 20.0; SPSS, Inc). Bivariate analysis was used to assess risk factors among cases and controls. Binary logistic regression analysis was done to evaluate the effects of risk factors which showed significant association during risk factor assessment according to menopausal status in breast cancer females.

## Results

*Patients*

A total of 271 female breast cancer patients were recruited in the study. 83.4% of the patients had sporadic breast cancer. Majority of the patients were diagnosed at stage II (52.2%) and stage III (23%) breast cancer. Of these, 47.5 % patients were triple negative for the receptor status.

*Patients and Controls*

271 healthy female individuals matched for age from the same geographical area were recruited as controls in the present study. The mean age of patients was 47.73±10.22 years and the mean age of controls were 47.22±10.46 years. 78.6% subjects belonged to rural area. They were homemakers and indulged in domestic chores along with lending help in agricultural activities. The remaining proportions of subjects belonged to urban (13.6%) and suburban (7.8%) areas and were mainly homemakers. 92.6% females were vegetarians. Table 1 shows the comparison of demographic, reproductive and anthropometric characteristics of patients and controls. Bivariate analysis for risk factors (Table 2) showed a lower risk (OR=0.65, 95% CI 0.43-0.99, p=0.04) in obese cases with BMI≥25 kg/m^2^ as compared to subjects with normal BMI. For other factors there was no significant association seen in either cases or controls.

*Pre- and Postmenopausal breast cancer females*

Table 3 shows the risk factor analysis of breast cancer patients according to menopausal status. Out of 271 breast cancer patients, 103 (38%) were premenopausal and 158 (58.3%) were postmenopausal. Parameters which provided risk for postmenopausal breast cancer were BMI (overweight: OR=0.39, 95% CI 0.17-0.92, p=0.03; obesity: OR= 0.26, 95% CI 0.13-0.52, p=0.00), WC (OR=0.17, 95% CI 0.05-0.52, p=0.00) and WHtR (p=0.02). WHR was not associated with breast cancer risk. However, parity and breastfeeding were significantly associated with premenopausal breast cancer. Premenopausal women with 3 or less than 3 children had a higher risk (OR=5.54, 95 % CI 2.75-11.19, p=0.00) than postmenopausal women when compared to women with more than 3 children. In all the 3 subgroups of breastfeeding for duration less than 2 years, premenopausal women were seen to be at significantly higher risk as compared to postmenopausal women. To assess the effects of parity and breastfeeding in premenopausal breast cancer females, binary logistic regression analysis was done (Table 4), which revealed that parity (≤3) substantially increased the risk for breast cancer (OR=4.80, 95% CI 2.34-9.85, p=0.00) in premenopausal women.

To evaluate the effect of menopausal status amongst cases and controls on breast cancer risk, statistical analysis was performed (data not shown) but no substantial association was found.

**Table 1:** Characteristics of Breast Cancer Cases and Matched Controls

Characteristic	Cases (n=271)	Controls (n=271)
Age in years (Mean± SD) (Range)	47.73±10.22 28-70	47.22±11.46 26-70
Age at Menarche (Mean± SD)	14.97±1.47	14.94±1.28
Age at Menopause (Mean± SD)	44.66±4.35	45.32±5.13
Age at first live birth (Mean± SD)	22.39±2.84	22.22±2.79
Menopausal status		
Pre-menopausal n(%)	103 (38)	117 (43.17)
Post-menopausal n(%)	158 (58.3)	141 (52.03)
BMI (Mean±SD)	25.66 ±5.19	26.58 ±4.58
WC in cm (Mean±SD)	96.43 ±12.33	96.38 ±11.65
WHR		
Normal (0.81)	2	0
Adiposity (0.81)	269	271
WHtR		
Normal (<0.5)	4	4
Adiposity (≥0.5)	267	267

**Table 2:** Bivariate Analysis of Breast Cancer Patients and Healthy Controls According to Risk Factors Assessed

Parameter	Categories	Cases	Controls	p-value	OR (95% CI)
BMI (kg/m^2^)	Normal (18.5-22.9) Overweight (23-24.9) Obese (≥25)	70 41 143	53 45 167	0.19 0.04	1 (ref) 0.69(0.40-1.2) 0.65 (0.43-0.99)
WC (cm)	Normal (<80cm) Central Obesity (≥80cm)	18 253	16 253	0.74	1 (ref) 0.89 (0.44-1.78)
WHR	Normal (0.81) Adiposity (0.81)	2 269	0 271	0.49*	1 (ref) ND
WHtR	Normal (<0.5) Adiposity (≥0.5)	4 267	4 267	1.00*	1 (ref) 1 (0.25-4.04)
Parity	≤3 >3	193 78	187 84	0.57	1.11(0.77-1.61) 1 (ref)
Breastfeeding	≤6 months >6months or ≤1 year >1 year or ≤ 2 years >2 years	30 11 30 199	24 6 32 190	0.54 0.27 0.69	1.19 (0.67-2.12) 1.75 (0.64-4.83) 0.90 (0.52-1.53) 1 (ref)

CI, Confidence Interval; *, Fischer’s Exact test; ND, Not Defined

**Table 3:** Risk Factor analysis of Breast Cancer Patients According to Menopausal Status

Parameters	Categories	Premenopausal	Postmenopausal	p-value	OR (95% CI)
BMI (kg/m^2^)	Normal (18.5-22.9) Overweight (23-24.9) Obese (≥25)	32 17 45	17 23 92	0.03 0.00	1 (ref) 0.39 (0.17-0.92) 0.26 (0.13-0.52)
WC	Normal (<80cm) Central Obesity (≥80cm)	14 89	4 154	0.00*	1 (ref) 0.17 (0.05-0.52)
WHR	Normal (0.81) Adiposity (0.81)	2 101	0 158	0.16	1 (ref) ND
WHtR	Normal (<0.5) Adiposity (≥0.5)	4 99	0 158	0.02*	1 (ref) ND
Parity	≤3 >3	92 11	95 63	0.00	5.54 (2.75-11.19) 1 (ref)
Breastfeeding	≤6 months >6months or ≤1 year >1 year or ≤ 2 years >2 years	15 8 15 64	11 3 14 128	0.02 0.01* 0.05	2.73 (1.19-6.28) 5.33 (1.37-20.79) 2.14 (0.98-4.71) 1 (ref)

OR, Odds Ratio; CI, Confidence Interval; *, Fischer’s Exact test; ND Not Defined

**Table 4:** Binary Logistic Regression Analysis of Breast Cancer Patients

Parameter	Categories	p value	Adjusted OR (95% CI)
Parity	≤3 >3	0.00	4.8 (2.34-9.85) 1 (ref)
Breastfeeding	≤6 months >6months or ≤1 year >1 year or ≤ 2 years >2 years	0.23 0.05 0.22	1.65 (0.73-3.72) 4.05 (0.99-16.45) 1.66 (0.73-3.77) 1 (ref)

OR, Odds Ratio; CI, Confidence Interval

## Discussion

This study intends to assess the epidemiological risk factors for breast cancer in females as its frequency is increasing in adjoining areas of Amritsar city of Punjab, India (personal communication, Rotary Cancer Hospital, Sri Guru Ram Das Institute of Medical Education and Research, Vallah, Amritsar, Punjab). 

In the present case-control study, the control females were matched for age with the breast cancer females. On compiling the results, both cases and controls showed similarity in the characteristics like age at menarche, age at menopause and age at first live birth indicating that the cases and controls had similar menstrual and reproductive history. By controlling for age in the present study, we had aimed to assess the independent role of other factors that predispose the females to breast cancer risk in our study population inhabiting the Amritsar region of Punjab, India. As the age at menopause was self-reported by women, chances of recall bias cannot be ruled out. The data from breast cancer patients was collected from a single tertiary level hospital (Sri Guru Ram Das Institute of Medical Education and Research, Vallah, Amritsar, Punjab) therefore the sampling bias in cases cannot be completely ruled out. However, the control samples were randomly selected. 

The population in state of Punjab represents a middle income group where carbohydrate intake is high, making overweight and obesity rampant in the state (Sidhu and Kaur, 2005). In the present study there was no significant difference in any of the obesity associated parameters in patients and controls. Anthropometric factors like WC, WHR and WHtR revealed that majority subjects, whether cases or controls, fell into the obese category. However, the comparison among pre- and post menopausal breast cancer females, in the present study, showed that overweight (BMI≥23 kg/m^2^) and obesity (BMI≥25 kg/m^2^) were associated with postmenopausal breast cancer risk. Alongwith high BMI, high WC (≥80cm) and WHtR (≥0.5) were also associated with postmenopausal breast cancer risk. 

In overweight and obese postmenopausal women androstendione and testosterone get aromatized to estrogens in the adipose tissue subsequently leading to a rise in estrogen levels (Morris et al., 2011). Additionally, abundance of adipose tissue in obese women further causes higher endogenous synthesis of estrogens (Neuhouser et al., 2015). Leptin, which is present in higher levels in overweight and obese persons as compared to normal weight individuals (Hursting et al., 2012), has also been known to increase estrogen levels (Geisler et al., 2007). All these factors cumulatively predispose overweight and obese postmenopausal women to a higher risk of developing breast cancer. Some studies have found a positive relationship between obesity and risk of breast cancer development in postmenopausal women (Zhao et al., 2014; Borghesan et al., 2016) while other studies did not find any association (London et al., 1989; Feigelson et al., 2004). The association of BMI with premenopausal BC has also been reported to vary by ethnicity, with a positive association seen among Asian women, and an inverse association among Africans and Caucasians (Renehan et al., 2008; Amadou et al., 2013). A higher BMI has been consistently shown to be associated with increased risk of postmenopausal BC (Amadou et al., 2013). More frequent anovulatory menstrual cycles and lower estrogen concentrations have been known to lower breast cancer risk among obese premenopausal women (Potischman et al., 1996).

In the present study shorter duration of breastfeeding was seen to be an important risk factor for premenopausal breast cancer (Table 3), however, number of subjects who have breastfed for the duration of > 6months or ≤ 1year was too low to have statistical significance. In India, majority of the parous women have been breastfeeding in the past but this trend seems to be diminishing in the younger generation owing to the lifestyle changes. This can be clearly seen from our data as the patient group which has breastfed for longer duration (>2 years) was older postmenopausal females (82.1%) as compared to the younger premenopausal females (62.75%). This deviation in our data could have also occurred probably because more subjects were postmenopausal (58.3%) as compared to premenopausal (38%). Previous case-control studies from other regions of India, like- Delhi (Kaur et al., 2011), Nagpur (Meshram et al., 2009), Puducherry (Balasubramaniam et al., 2013) and Central India (Rangwala et al., 2017), have reported an association of breast cancer with lower duration of breast feeding and parity in patients. In a study conducted on South Indian females (Gajalakshmi et al., 2009), duration of breastfeeding was found to reduce the premenopausal breast cancer risk but not the postmenopausal. However, a large pooled analysis to assess breastfeeding and breast cancer risk, with a higher number of studies reported from high-income countries, showed that the protective effect of breastfeeding was similar in both pre- and postmenopausal females (Beral et al., 2004). Other studies (Millikan et al., 2008; Palmer et al., 2014), however, have reported the role of history of breastfeeding and longer duration of breastfeeding in significantly reducing the breast cancer risk among young but not in older women which emphasizes the importance of breastfeeding in lessening the extent of parity-associated risk in young women. 

In the present study, lower parity (≤3) was seen to provide a significant risk for breast cancer in premenopausal women, independently (Unadjusted OR=5.54, 95% CI 2.75-11.19, p=0.00) as well as when adjusted for duration of breast feeding (Adjusted OR=4.80, 95% CI 2.34-9.85, p=0.00). The populations of Caucasian and Indoscythian mixed racial elements inhabit the state of Punjab in North India (Bhasin et al., 1992). In Caucasian women, higher numbers of full term pregnancies has been shown to lower the risk of breast cancer in postmenopausal women whereas in premenopausal breast cancer there was a slight decrease in the risk (Hall et al., 2005). Similar findings have been reported from the studies on African American women (Hall et al., 2005; Palmer et al., 2003). A few studies on Asian populations have laid emphasis on the association between parity and breast cancer risk in premenopausal women residing in Asia, but the findings have been equivocal (Lee et al., 1992; Nichols et al., 2005; Chollet-Hinton et al., 2016).

It has been demonstrated that there is an association of a brief increase in breast cancer risk with every full-term pregnancy, which is followed by a decrease in risk 10 to 15 years later (Kelsey et al., 1993; Lambe et al., 1994; Liu et al., 2002). Subjects included in the present study had first live birth around 22 years of age, so most women had 2 to 3 children before 35 years of age. Thus parity can be a probable cause of parity associated risk of breast cancer in the premenopausal women or it may be associated with risk reduction in the postmenopausal women subjects, of our study. It has been hypothesized that harmful effect of pregnancy may be linked to inflammatory/immune system processes occurring during postpartum involution (Schedin, 2006). Involution embodies processes like wound healing and immunesuppression, both of which are identified as pretumorigenic. Breastfeeding may lower the risk from full-term pregnancy by extending the time between pregnancy and involution (Palmer et al., 2011). The number of premenopausal women who have breast fed for longer duration (more than 2 years) in our study, is much less. Therefore, this group is devoid of the protective effect of breastfeeding. Obesity, as already stated, is present in all the subjects of current study. All these factors cumulatively are suggestive of premenopausal breast cancer risk in the present study.

Dietary habits with high carbohydrate and fat intake in Amritsar region make overweight and obesity common, increasing obesity associated cancer risk for all women. Our study observed that firstly, obesity increases the risk in postmenopausal women and secondly, lesser parity provides considerable risk of breast cancer to premenopausal women represent a younger age group which seems to follow a trend of having less offspring, have a lifestyle with diminishing trend of physical activity and breastfeeding. Thus obesity, parity associated breast cancer risk and reduced breastfeeding cumulatively predispose the premenopausal women of this region to higher risk of breast cancer. We suggest that further longitudinal studies with larger sample sizes must be carried to validate our findings so that preventive measures can be adopted in the state. 
